# Increasing the Therapeutic Efficacy of Extracellular Vesicles From the Antigen-Specific Antibody and Light Chain Perspective

**DOI:** 10.3389/fcell.2021.790722

**Published:** 2021-11-24

**Authors:** Katarzyna Nazimek, Krzysztof Bryniarski

**Affiliations:** Department of Immunology, Jagiellonian University Medical College, Krakow, Poland

**Keywords:** antibody, biotherapeutics, exosomes, extracellular vesicles, treatment efficiency

## Abstract

Due to their exceptional properties, extracellular vesicles (EVs) receive special attention as next generation biotherapeutics and vehicles for drug delivery. However, despite having many advantages over cell-based therapies, EVs usually exert lower therapeutic efficacy. This results from a number of hurdles that are faced by the EV-based approaches. Administered EVs could be rapidly cleared by the mononuclear phagocytes as well as can randomly distribute within various tissues, making tissue penetration and cell targeting insufficient. However, recent research findings imply that these limitations could be overcome with the use of antigen-specific antibodies and light chains. Major histocompatibility complex (MHC) class II-expressing EVs have been shown to form aggregates after co-incubation with antigen-specific antibodies, which greatly enhanced their biological efficacy. On the other hand, EVs could be coated with antibody light chains of chosen specificity to direct them towards desired target cell population. Both findings open up a promising perspective to achieve the highest efficacy of the EV-based approaches. Herein we discuss the opportunities for enhancing extracellular vesicle’s biological activity by using specific antibodies and light chains in the context of the challenges faced by such therapeutic approach.

## Introduction

**FIGURE 1:**
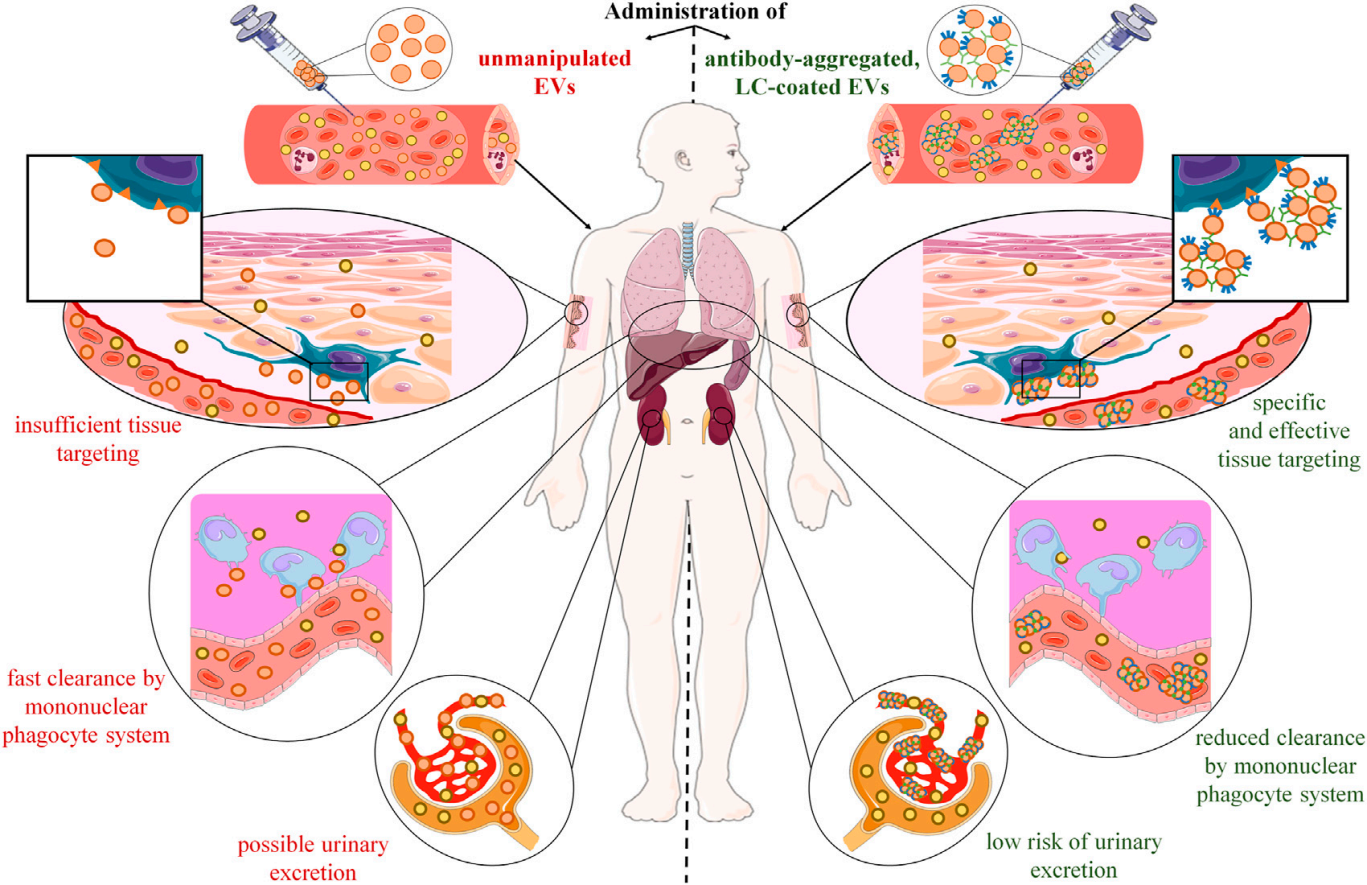
Postulated advantages of extracellular vesicle (EV) aggregation with antigen-specific antibodies and coating with antigen-specific light chains (LC). After systemic administration, therapeutic EVs disperse and mix with their counterparts in the circulation, from which they pass into the tissues. However, their tissue distribution is at least partly random with the preferential accumulation in mononuclear phagocyte-enriched organs, such as liver, spleen, and lungs, where they are rapidly cleared by macrophages. In addition, injected EVs could likely be excreted in urine due to their putative ability to penetrate kidney’s glomerular filtration barrier. Altogether, these hurdles make desired tissue penetration and cell targeting insufficient to induce the expected therapeutic effect. However, recent research findings showed that these limitations could be overcome with the use of antigen-specific antibodies and light chains. Incubating EVs with antigen-specific IgG antibodies leads to their aggregation, which enhances their biological efficacy by increasing the amount of EVs that target desired cell population, and by lowering the risk of urinary excretion. In addition, coating EVs with antigen-specific LCs directs them towards target cells, which augments the selectivity of tissue targeting, and limits the unwanted clearance by phagocytes.

Extracellular vesicles (EVs) encompass all classes of lipid-membrane vesicles that differ in the formation pathway but are then released by virtually all cells to their surrounding microenvironment (Yáñez-Mó et al., 2015; Pironti et al., 2021). As a newly discovered mood of intercellular communication, they currently receive special attention as next generation biotherapeutics with likely very limited adverse effects of administration (Kalluri and LeBleu, 2020), and multiple advantages over synthetic liposomes (Vader et al., 2016; Nazimek and Bryniarski, 2020b). Accordingly, EVs, exosomes especially, are considered promising vehicles for drug delivery due to their biocompatibility and exceptional stability in biological fluids (Akuma et al., 2019). In this aspect, however, it should be stressed that EVs themselves are very complex and thus their components should be taken into consideration as additional active drug constituents (Lener et al., 2015). Furthermore, EVs could be used by pathogens for infection spreading, and thus one can speculate that they may transfer virulence factors, which has to be taken into consideration while manipulating EVs for therapeutic applications (Pironti et al., 2021). On the other hand, attempts to solve the challenges of stem cell therapy allowed to discover that EVs are the main paracrine factors that actually mediate the effects induced by administration of their parental cells (Johnson et al., 2021; Wang et al., 2021). However, despite having many advantages over such approaches, EV-based therapeutics usually achieve lower or almost the same therapeutic efficacy than releasing cells (Kalluri and LeBleu, 2020). Our current research findings suggest that some of these limitations could be overcome by aggregating EVs with antigen-specific antibodies and by increasing the specificity of cell targeting with antibody light chains (LCs). As discussed below, such approaches offer a promising perspective in enhancing EV’s therapeutic activity (Figure 1).

## “Poor is the Pupil Who Does not Surpass his Master”^1^

Recent studies and clinical trials uncovered a number of hurdles that are faced by the cell-based therapies. The widely described obstacles affecting the efficacy of stem cell therapies result mainly from a lack of standardized treatment procedures, low percentage of cells that reach the desired tissue/organ, the poor survival of engrafted cells that often rapidly undergo apoptosis in targeted tissues, and finally from the diversity of yielded cell populations between individual donors (Hassanzadeh et al., 2021; Johnson et al., 2021; Wang et al., 2021). In addition, stem cell therapies raise concerns about possible adverse events of the treatment that are associated with the risk of tumorigenesis, the possibility of disrupted or abnormal maturation of infused cells as well as with their eventual differentiation in undesirable tissue (Lukomska et al., 2019). On the other hand, the main obstacle related to immunotherapy with dendritic cells results from the risk of their phenotype switching under the influence of the tissue microenvironment, especially at the site of tumorigenesis (Gardner et al., 2020). The risk of losing the desired phenotype may also apply to bone marrow-derived macrophages (Cao et al., 2014), and likely other types of therapeutically administered living cells. Finally, cytokine release syndrome is a major severe complication of the very promising anti-tumor therapy with chimeric antigen receptor (CAR)-T cells that results from the uncontrolled activation of recipient cells by transferred CAR-T lymphocytes (Cosenza et al., 2021).

Abovementioned obstacles and concerns prompted researchers to search for safer alternatives that could efficiently replace the living cells for therapeutic purposes. Consequently, EVs released by these cells become considered main promising candidates for cell-free therapies (Tang et al., 2015; Johnson et al., 2021; Wang et al., 2021; Yao et al., 2021).

Along these lines, unlike releasing cell, EVs do not contain all components of cellular machinery required for proper functioning of the living cell, but still they can substitute for its activity. The latter is likely possible due to the contained cargo, which enables EVs to perform a specific function. This assumption is supported by the fact that the cargo is sorted into EVs, exosomes especially, in a very sophisticated manner, and represents a unique content of proteins, RNAs, and lipids, usually differing from the parental cell (Wei et al., 2021). Thus, stem cell-derived EVs cannot differentiate but can promote immune tolerance (Wang et al., 2021), and tissue regeneration (Johnson et al., 2021) instead of tumorigenesis (Lukomska et al., 2019), since they do not carry full genomic DNA material. Moreover, EVs can carry and present antigens as do dendritic cells, but they are unable to switch their phenotype (Yao et al., 2021). Finally, EVs can induce antigen-targeted cytotoxicity against cancer cells without the risk of cytokine release syndrome development (Tang et al., 2015).

It is worth noting that, although EV-based strategies also do not have standardized protocols yet, other concerns and hurdles are either not relevant or could be easily avoided, since EVs are much more manipulable than their parental cells. The latter EV feature together with still expanding knowledge about their biology and functions opened up a new research area attempting both to improve the properties of cell-derived EVs and to design and manufacture their artificial counterparts (García-Manrique et al., 2018) in order to design the most appropriate therapeutic modality, for example in personalized medicine. Such possibilities support the consideration of either manipulated or engineered EVs as next generation biotherapeutics with almost unlimited therapeutic activities and indications that would be easy to produce, handle, and distribute (Johnson et al., 2021). Obviously, as discussed below, selecting the best cellular source of EVs for each considered application is the first crucial step on the way to personalize the EV-based therapeutics (Campanella et al., 2019; Logozzi et al., 2021a). However, before it will happen, researchers have to learn how to improve EVs to surpass both their current therapeutic efficacy and parental cell activities.

## Challenges in Therapeutic Application of Extracellular Vesicles

Among other concerns, EV-based approaches avoid the risk of gene mutation, uncontrolled cell division and differentiation as well as immune rejection (Hassanzadeh et al., 2021). Therefore, EVs are considered safer, more controllable as well as much less immunoreactive and immunogenic than the therapeutically administered cells (Buschmann et al., 2021). Other undoubted advantages over the cell-based therapies result from EV’s high stability and resistance to harsh conditions *in vivo,* biocompatibility, ability to cross physiological barriers as well as from their rapid uptake into tissues (Johnson et al., 2021). These features entail a significant increase in interest in the possibility of their therapeutic application, which is still fraught with many challenges (Claridge et al., 2021).

At present, the challenges of standardizing the protocols of EV’s generation, characterization, loading with a cargo, dosing and administration are brought to the fore to meet good manufacturing practice requirements (Lener et al., 2015; Buschmann et al., 2021; Thakur et al., 2021). Obviously, choosing the right EV source (i.e., either proper releasing cell subtype or biological fluid), the best loading strategy and the optimal dose administered via the most efficient route would produce the highest efficacy of treatment. But very likely these choices would vary across the specific clinical indications or even patients. The current state-of-the-art technologies and methodologies have been widely summarized elsewhere (Akuma et al., 2019; Doyle and Wang, 2019; Li et al., 2019; Gowen et al., 2020; Klyachko et al., 2020; Gupta et al., 2021; Johnson et al., 2021). However, the use of EV-based therapeutics requires still a very flexible approach for optimization, as it is built on the basis of constantly growing knowledge and clinical evidences.

Although the stem cell therapy achieved promising efficacy with minimal number of reported adverse events in patients with immune-related and inflammatory diseases, stem cell-derived EVs have recently emerged as the easier-to-use treatment modality (Wang et al., 2021) with no risk of stem cell-related coagulopathy (Askenase, 2020) and occlusions (Zhang et al., 2014).

On the other hand, despite promising results in basic and preclinical studies, EVs showed lower than expected therapeutic activity in some cancer clinical trials. Along these lines, dendritic cell-derived EVs demonstrated low clinical efficacy as maintenance immunotherapy in patients bearing inoperable but not progressive non-small cell lung cancer (Besse et al., 2016). This could be due to the fact that EVs were collected from immature dendritic cells and then administered to patients with advanced cancer that likely had impaired immunoreactivity (Yao et al., 2021). This clearly shows that the immune status of patients in target group has to be taken into account while designing appropriate EV-based drug or vaccine.

Another problem is related to pharmacodynamics and pharmacokinetics of therapeutically administered EVs, insufficient tissue penetration and cell targeting especially. Among other possible causes, these limitations could be due to both the dispersion of EVs in biological fluids, and their rapid clearance from circulation by the mononuclear phagocyte system (Figure 1).

Accordingly, after systemic administration, EVs can disperse fast in lymph and blood plasma, where they mix with their counterparts from other cellular sources (Nazimek et al., 2016) and form colloid suspension. Interestingly, EV’s colloidal stability in biological fluids seems to be affected by their natural tendency to aggregate (Hood et al., 2014). It is worth noting that EV’s aggregation could be enhanced by electroporation (Hood et al., 2014), which may impede their further processing for therapeutic application. However, one can speculate that formation of such “colloid aggregates” may increase EV’s half-life in circulation by dampening their random passage to tissues. In addition, aggregated EVs would be less likely to overcome the glomerular filtration barrier and become excreted in urine (Erdbrügger et al., 2021). Moreover, it can be hypothesized that EV’s aggregation together with directing them towards desired target tissue or cell population will greatly enhance their therapeutic efficacy. As discussed below, our results showed that this could be achieved by incubating EVs with antigen-specific antibodies (Nazimek et al., 2021) (Figure 1).

On the other hand, injected EVs were found to be rapidly cleared by macrophages of the mononuclear phagocyte system. Therefore, they preferentially accumulate in mononuclear phagocyte-enriched organs, such as liver, spleen, and lungs (Chen et al., 2021). Furthermore, resident macrophages are considered to clear EVs also in other tissues. For instance, microglia can phagocytose and thus remove EVs from the brain parenchyma (Schnatz et al., 2021). Thus, fast clearance by macrophages not only hampers EV’s delivery to desired tissue and target cell population, but also may either affect the expected therapeutic action or cause some side effects. The latter could be observed if EV’s are not cleared in an immunologically silent process but instead when it leads to macrophage activation. Such activation most likely depends on the EV-contained cargo and the state of the donor cell, as discussed recently in the case of microglia (Schnatz et al., 2021). Additionally, one can speculate that macrophages in inflamed tissue are possibly more prone to undergo unexpected activation after EV’s engulfment. This seems to particularly apply to the EV-based therapeutics targeting the cells at the site of tissue injury or ongoing inflammation. However, the unwanted clearance could likely be limited by decorating therapeutic EVs with molecules providing “don’t eat me” signals (Belhadj et al., 2020), especially in a combination with strategy increasing the specificity and selectivity of cell targeting. And, following the commonly known slogan “simplicity is the ultimate sophistication,” antigen-specific antibodies and their derived LCs appear most promising candidates for the latter strategy (Figure 1).

## Antigen-specific Antibodies and Light Chains in the Fight to Enhance Extracellular Vesicles Therapeutic Efficacy

In *in vivo* conditions, EVs gain most of their attributes at the time of the release by parental cell. However, some of them can be acquired later in the extracellular space. Accordingly, recent study demonstrated the spontaneous formation of a protein corona around EVs in blood plasma (Tóth et al., 2021). Very likely the acquired proteins impact EV’s properties and activity, which requires further investigation (Tóth et al., 2021). Similarly, our observations suggested the ability of EVs to bind freely circulating miRNAs (Bryniarski et al., 2015). This could likely be applied in small interfering RNA (siRNA) delivery, as can recently developed EV-liposome hybrid nanoparticles (Evers et al., 2021). These findings also imply that EVs may constitute physiological nanocarriers for removing other molecules from the circulation. Conversely, proteins adhered to EV membrane may redirect the vesicles towards other target.

**FIGURE 2:**
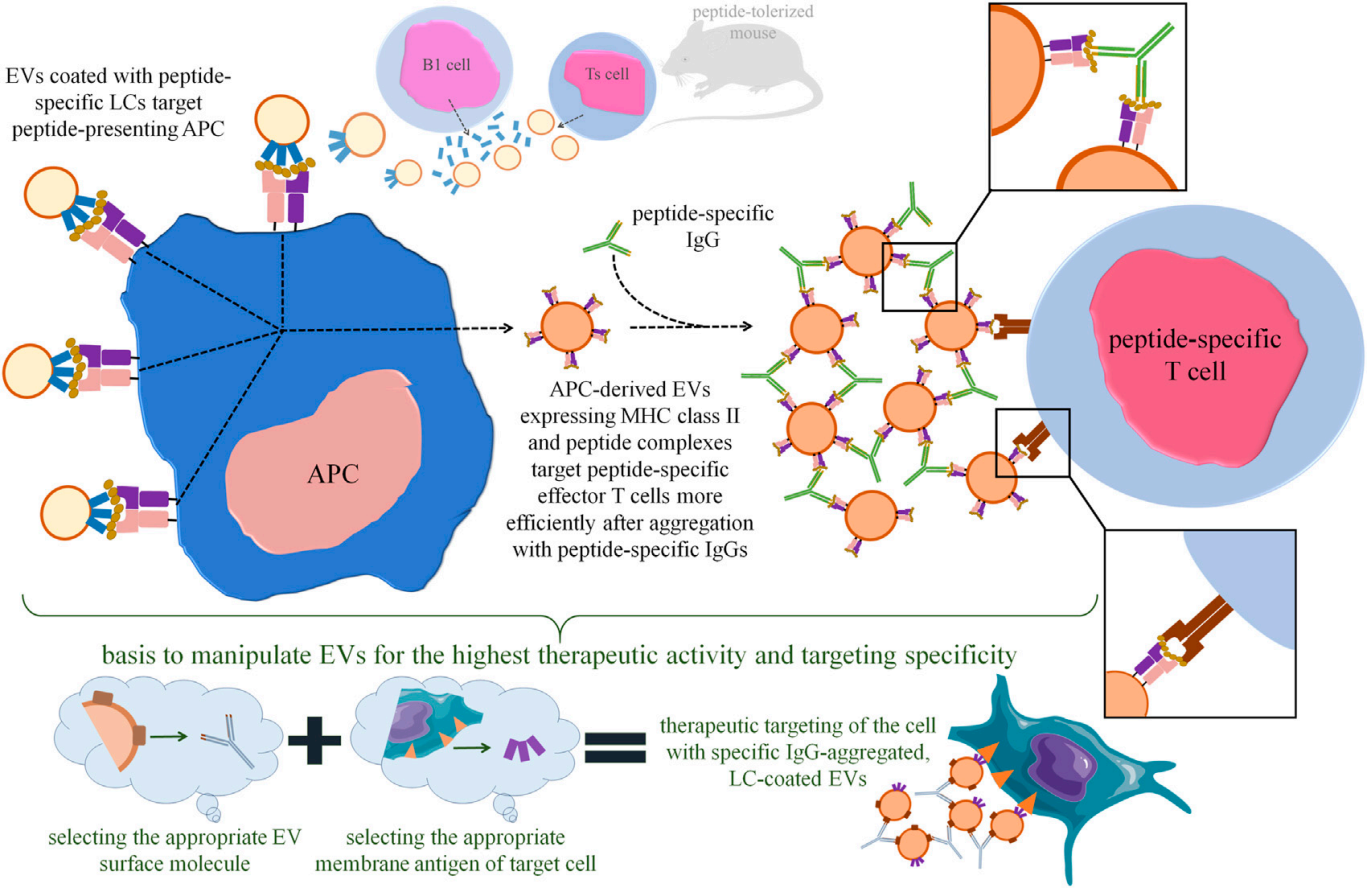
Perspectives in increasing the therapeutic efficacy of extracellular vesicles (EVs) with the use of antigen-specific antibodies and light chains (LCs). Our research findings demonstrated that tolerizing mice with antigen-coupled syngeneic erythrocytes activates suppressor T (Ts) cells to release EVs containing immune regulatory miRNA-150. These EVs are then coated with B1 lymphocyte-secreted, antigen-specific LCs, which allows EVs to specifically bind the antigenic peptide presented by antigen-presenting cell (APC). Importantly, Ts cell-derived EVs could also be *in vitro* coated with LCs of chosen specificity by simple co-incubation. The Ts-cell-EV-targeted APC releases its own EVs expressing major histocompatibility complex (MHC) class II and antigenic peptides that target peptide-specific effector T cells by interacting with T-cell receptor (TCR). Incubation of APC-derived, MHC-expressing EVs with peptide-specific IgG antibodies leads to vesicle aggregation, which greatly enhances their immune suppressive activity by increasing the amount of EVs targeting each effector T cell. Based on these observations, one can assume that therapeutic EVs in general could be manipulated to achieve the highest activity by aggregating them with IgG specific for the selected EV surface molecule, and then by selectively directing them towards target cell with LCs specific for the selected cell surface antigen.

Along these lines, we found that mouse suppressor T (Ts) cell-derived EVs are coated with B1a cell-secreted antibody LCs (Bryniarski et al., 2013; Wąsik et al., 2019; Nazimek et al., 2020). Detailed investigation of this surprising observation allowed us to discover that LC coating ensures the antigen-specificity of Ts cell-released EV’s action (Nazimek et al., 2018). Furthermore, coating EVs *in vitro* with LCs of chosen specificity enables the antigen-specific suppression of immune response (Bryniarski et al., 2013; Nazimek et al., 2018, 2020) (Figure 2). Finally, self-tolerant EVs become capable of suppressing hapten-induced contact hypersensitivity after co-incubation with hapten-specific LCs (Nazimek et al., 2019).

We proposed that LCs stick to EV membrane lipids and form multiple adjacent arrangements of high avidity (Nazimek et al., 2016), sufficient for binding to antigenic peptides presented by antigen-presenting cells (APCs) (Nazimek et al., 2020). These findings imply the important role of LCs in specific cell targeting by EVs (Nazimek and Bryniarski, 2020a), thereby increasing their therapeutic potential (Figure 2). Especially that LCs could be coated onto EVs by simple co-incubation, which is a technically easy approach. As mentioned above, protein corona can be acquired by EVs during similar co-incubation with plasma samples (Tóth et al., 2021). Thus, one can assume that the ability to become coated with proteins, including antigen-specific antibodies and LCs, is a general property of EVs that could be widely used for manipulating them for therapeutic purposes. It is also worth noting that the delivery of therapeutic antibodies by EVs can increase their stability and bioactivity, as recently shown in the case of immunoglobulin-bearing EVs produced by OKT3 hybridoma (Logozzi et al., 2021b).

Accordingly, considering the immune checkpoint therapy, one can assume that it could be reinforced by co-administration of EV-based therapeutics. In this case, decorating therapeutic EVs with antibodies specific for programmed death receptor-1 ligand (PD-L1) would direct them towards PD-L1-expressing tumor cells, while anti-cytotoxic T-lymphocyte-associated protein 4 (CTLA-4) antibodies would support regulatory T cell targeting (Nazimek and Bryniarski, 2020b). Hence, such combined strategy would allow to block immune checkpoints and induce EV-mediated therapeutic effect at the same time, as recently reported (Fan et al., 2021). However, the possibility that therapeutic EVs decorated with anti-PD-L1 antibodies could be bound by PD-L1-expressing tumor EVs to hamper tumor cell targeting cannot be excluded as well, by analogy to the therapies with immune checkpoint inhibitors (Nazimek and Bryniarski, 2020b). Especially that tumor cells are known to potently release EVs into circulation (Logozzi et al., 2021c).

On the other hand, almost 20 years ago it was observed that cross-linking of major histocompatibility complex (MHC) class II-expressing EVs with latex beads greatly augments their biological effectiveness (Vincent-Schneider et al., 2002). Similar enhancement was recently demonstrated by us after aggregating MHC class II-expressing EVs with antigen-specific antibodies (Nazimek et al., 2021). These findings imply that EV’s aggregation may increase not only their half-life in circulation, as mentioned above, but also the amount of vesicles targeting desired cell population (Figures 1, 2). The latter fits in with the consideration of the optimal EV’s dosing at a single cell scale and at the body scale (Gupta et al., 2021). Proposed by us EV’s aggregation with antigen-specific antibodies takes an advantage over the latex beads, since antibodies could be safely degraded intracellularly, could limit the unwanted clearance of EVs by phagocytes, and may increase the specificity of cell targeting (Nazimek et al., 2021). Besides, one can assume that EV’s could be similarly aggregated with antibodies directed against their surface proteins, which greatly extends the application spectrum of this phenomenon (Figure 2). However, chosen surface molecules bound by aggregating antibodies cannot be involved in EV’s cellular uptake to not disturb this process. The latter is implied by the observation that the attempts to use anti-CD9 antibodies abolished the immune activity of EVs (Nazimek et al., 2021).

Additionally, some other aspects of this phenomenon require further investigation. Technically, EV’s aggregation could be controlled *in vitro* with transmission electron microscopy and nanoparticle tracking analysis after standardizing and optimizing the protocols of obtaining the aggregates. Whereas the methods to track the stability and bioactivity of EV’s aggregates in *in vivo* conditions have yet to be developed. Moreover, since aggregation was firstly proved for MHC class II-expressing EVs, the possible MHC-restriction of their activity has to be elucidated with regard to the possibilities to use EVs from allogeneic donors. On the other hand, one can speculate that autologous dendritic cell-derived EVs loaded *in vitro* with dedicated peptides in a direct or indirect manner (Morse et al., 2005) could be considered optimal for aggregation to greatly enhance their vaccine-like activity for instance in cancer immunotherapy (Yao et al., 2021).

## Conclusion

Last 2 decades have resulted in tremendous advances in the knowledge of the exceptional functions of EVs, which made them promising candidates for various therapeutic applications. Numerous current research findings have provided the basis for attempting to address the challenges faced by such therapies. Herein discussed possibilities to enhance EV’s biological activity and specifically direct them towards desired cells with the use of antibodies and LCs constitute crucial steps forward to achieve the highest efficacy of EV-based therapeutics.

## Data Availability

The original contributions presented in the study are included in the article/supplementary material, further inquiries can be directed to the corresponding author.

## Footnotes

1 Leonardo Da Vinci (quote translated by Jean Paul Richter)
